# Contribution of Ex-Situ and In-Situ X-ray Grazing Incidence Scattering Techniques to the Understanding of Quantum Dot Self-Assembly: A Review

**DOI:** 10.3390/nano10112240

**Published:** 2020-11-12

**Authors:** Vishesh Saxena, Giuseppe Portale

**Affiliations:** 1 Zernike Institute for Advanced Materials, University of Groningen, Nijenborgh 4, Groningen AG 9747, The Netherlands; v.saxena.1@student.rug.nl; 2 Macromolecular Chemistry and New Polymeric Material, Zernike Institute for Advanced Materials, University of Groningen, Nijenborgh 4, Groningen AG 9747, The Netherlands

**Keywords:** GISAXS, GIWAXS, quantum dot, self-assembly, grazing incidence scattering, superlattices, in-situ

## Abstract

Quantum dots are under intense research, given their amazing properties which favor their use in electronics, optoelectronics, energy, medicine and other important applications. For many of these technological applications, quantum dots are used in their ordered self-assembled form, called superlattice. Understanding the mechanism of formation of the superlattices is crucial to designing quantum dots devices with desired properties. Here we review some of the most important findings about the formation of such superlattices that have been derived using grazing incidence scattering techniques (grazing incidence small and wide angle X-ray scattering (GISAXS/GIWAXS)). Acquisition of these structural information is essential to developing some of the most important underlying theories in the field.

## 1. Introduction

Nanoparticles (NPs) have been the subject of immense research in the last few decades, focusing on understanding their behavior at the atomic scale. NPs are being used widely in almost all fields of research such as medicine, agriculture, food packaging, sensing devices, coatings and many more [1,2,3,4,5,6,7,8,9,10,11,12,13]. The wide use of NPs comes from their intrinsic amazing property of high surface area to volume ratio which makes them exhibiting interesting chemical, physical, electronic, magnetic properties which differ from the bulk scale ones. A special class of NPs are quantum dots (QDs), which are nm-sized particles with a tunable band gap with size variation [14,15]. QDs are zero dimension nanoparticles. Electrons residing on the dot occupy quantized energy levels. The degree of confinement in QDs is size dependent and it affects the occupied density of states which causes changes in electrical and optical properties [16]. Thanks to the peculiar physical properties, QDs have been long since used as fluorescence materials [17,18,19,20] and in optoelectronics applications [21,22,23,24].

In their solid form, QDs can self-assemble forming ordered structures, called superlattices, whose formation is under extensive study [25,26]. The most common form of self-assembly is driven by solvent evaporation (drop casting), wherein the solvent is evaporated at a calculated rate and the QDs begin to self-assemble to minimize the surface energy [27,28,29,30,31,32].

Gaining an insight into the growth mechanism of QD superlattices can provide important information regarding the growth kinetics, the ordering type of the assembled structures, the crystallographic orientation of the QDs during the growth, etc. Grazing incidence small and wide angle X-ray scattering (GISAXS/GIWAXS) are surface sensitive techniques which best suit the analysis of the processes mentioned above, when compared microscopy techniques such as transmission electron microscopy (TEM), scanning electron microscopy (SEM) and atomic force microscopy (AFM). By performing GISAXS/GIWAXS analysis using X-rays from synchrotron sources, in-situ analysis with high spatial–temporal resolution can be carried out. Moreover, GISAXS and GIWAXS analysis provides information about the crystallographic orientations at the surface and in the bulk of the assembled superlattices, not only on a small local region as in TEM, SEM or AFM, but on a mm^2^ scale. Additionally, structural modification induced by chemical reactions such as ligand exchange can be followed in-situ during drying.

In this review, we present an understanding of the growth of ordered superlattices via the self-assembly of quantum dots by reviewing different GISAXS/GIWAXS experiments carried out recently in this research field. We have taken nano-crystals (NCs) as quantum dots into consideration too in this review. This point is important to mention here, as in many papers authors have used the terms of quantum dots and nanocrystals interchangeably. In this review, we will focus on works performed on QDs of diameter in the range of 2–20 nm, as these QDs show the most interesting optoelectronic properties. The review is structured on the basis of different chemical nature of quantum dots.

## 2. Grazing Incidence Small and Wide Angle X-ray Scattering (GISAXS/GIWAXS) 

**Figure 1:**
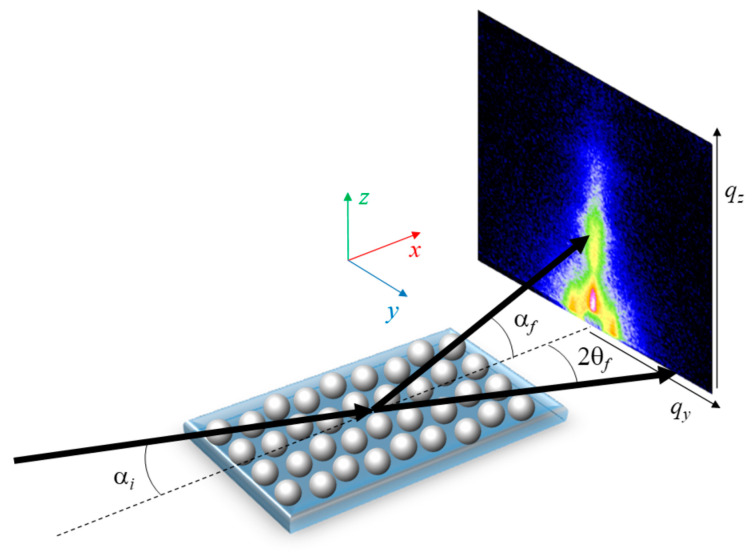
Schematic of a grazing incidence small and wide angle X-ray scattering (GISAXS/GIWAXS) experimental setup showing the incident ($\alpha_{i}$) and the scattered angles along the horizontal ($2\theta_{f}$) and vertical ($\alpha_{f}$) direction. X-rays are coming along the x direction.

Traditionally, X-ray scattering experiments are performed in transmission geometry (sample placed at 90° with respect to the incoming X-ray beam). However, for nanoparticles supported on thick, flat substrates such as QD superlattices, this geometry is not an option. Thus, the use of a grazing incidence (GI) geometry is obligatory. In the GI geometry, X-rays impact the sample at small incident angles (typically $\alpha_{i}$ < 0.5°) around or above the critical angle ($\alpha_{c}$) of the sample or of the substrate (Figure 1). Depending on if the detector is placed far away or close to the sample, a smaller or larger scattering angular range is probed, allowing it to cover the gracing incidence small angle X-ray scattering (GISAXS) or the gracing incidence wide angle X-ray scattering (GIWAXS) range, respectively. Typically, we distinguish between GISAXS (length-scales in the 1–1000 nm range) and GIWAXS (0.1–1 nm). Using two dimensional detectors, the scattering signal in the direction parallel (y direction) or perpendicular (z direction) to the substrate is collected at once (Figure 1).

The scattering vector components along the three spatial directions are defined as

(1) $$q=\{\begin{array}{c} q_{x}=\frac{2\pi }{\lambda }\left[\cos\left(2\theta_{f}\right)\cos\left(\alpha_{f}\right)-\cos\left(\alpha_{i}\right)\right]\\ q_{y}=\frac{2\pi }{\lambda }\left[\sin\left(2\theta_{f}\right)\cos\left(\alpha_{f}\right)\right]\\ q_{z}=\frac{2\pi }{\lambda }\left[\sin\left(\alpha_{f}\right)+\;\sin\left(\alpha_{i}\right)\right] \end{array}$$

The above Equations show the relation between $q_{x}$, $q_{y}$, $q_{z}$ (scattering vector components) and the incident angle ($\alpha_{i}$), the in-plane ($2\theta_{f}$) and the out-of-plane ($\alpha_{f}$) scattering angles [33]. $k_{0}$ is the wave vector defined by the X-ray wavelength. Please note that researchers in this field use Q or q interchangeably, but in this review we will use q wherever needed within the text.

When using GISAXS/GIWAXS for QD superlattices, some important advantages exist: (1) the penetration of X-rays in the substrate is quite limited when $\alpha_{i}\approx \alpha_{c}^{substrate}$, so the background is generally low; (2) the scattering intensity of the thin layer (or submonolayer) supported on the substrate can be greatly enhanced due to the minimum penetration in the substrate and maximum intensity of the evanescent wave when $\alpha_{i}\approx \alpha_{c}^{substrate}$; (3) as the footprint of the beam is as long as the length of the sample along the beam path (typically mm to cm), large scattering intensity is collected at the detector and statistically meaningful information can be obtained; (4) it is possible to run experiments in the second and sub-second timescales when using synchrotron radiation; (5) GISAXS/GIWAXS investigations at the solid–liquid, liquid–air and liquid–liquid interface can be performed. Moreover, information about the surface region, the bulk of the deposited film or even beneath the substrate surface, such as buried clusters of nanoparticles can be probed by adjusting the incident angle with respect to the sample and substrate critical angles.

## 3. PbS Quantum Dots

PbS quantum dots (or nanocrystals) have been studied extensively due to their application in organic solar cells allowing a high efficiency of 7.9%, [34] as electron blocking layers in solar cells, [35] and in photodetector [36]. Moreover, devices based on superlattices of colloidal PbS QDs are also being investigated [26,37].

The mechanism of superlattice formation via self-assembly of PbS QDs deposited onto silicon substrates via drop casting technique was studied by M. Corricelli et al. using both in-situ and ex-situ GISAXS/GIWAXS [28]. The effect of size and concentration of organic capped PbS QDs on the superlattice formation and the final superlattice structural ordering was studied. PbS QDs of 2.7 nm and 3.3 nm diameter were investigated at three different initial concentrations dispersed in toluene. In-situ GISAXS results revealed that the onset of superlattice formation for PbS QDs of 2.7 nm occurs earlier than the 3.3 nm PbS QDs.

**Figure 2:**
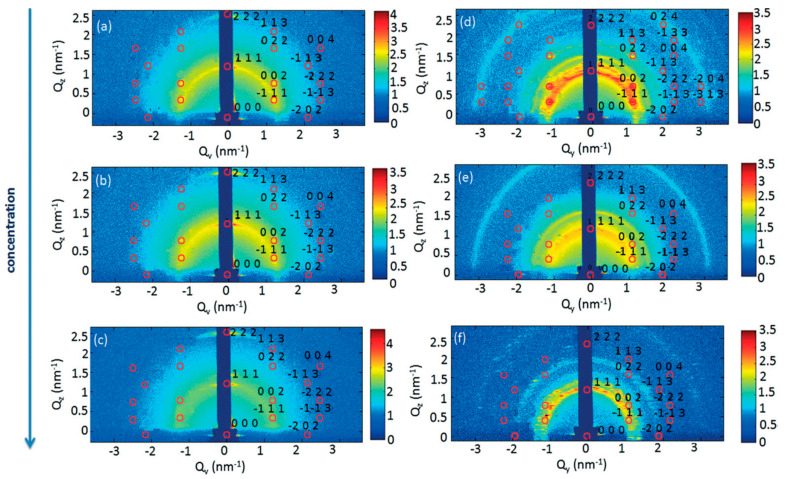
GISAXS patters for PbS_2.7_ (**a**–**c**) and PbS_3.3_ (**d**–**f**) indicating the process of formation of layered structures. Frames shown are at the end of the self-assembly process occurring during drying (30 min). Reprinted with permission from Ref. [28]. Copyright 2014, Royal Society of Chemistry.

As can be seen from Figure 2, there is formation of layered structures with a characteristic repeating distance of (111) and (002) *fcc* superlattice planes. There were no deviations reported in the GISAXS pattern from the *fcc* pattern. With increasing initial QD concentration it was observed that the GISAXS intensity reduces and there is a lower degree of assembly order.

This observation could be the result of entropic effects and repulsions due to coulomb potential becoming important when ensembles of QDs of such small sizes come close to each other as a result of increasing concentration. Ex-situ GISAXS measurements on these quantum dots showed that there was no change in the *fcc* structural ordering even after aging in air, suggesting good stability of the structures. Nevertheless, a contraction of the unit cell parameters was observed for both sets of PbS QDs (10% for PbS_2.7_ and 7% for PbS_3.3_) due to the oxidation of quantum dots in air. Ex-situ GIWAXS studies showed that the PbS_2.7_ were reported to be more spherical and not showing preferential ordering as compared to the PbS_3.3_ which showed (110) preferred orientational ordering.

**Figure 3:**
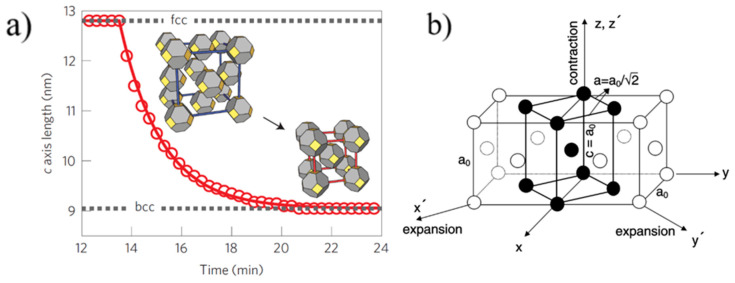
(**a**) Temporal contraction observed for 5.6 nm PbS quantum dots (QDs). (**b**) Bain distortion observed during the *fcc*–*bcc* transformation in martensite steel. (**a**): Reproduced with permission from Ref. [29]. Copyright 2016, Springer Nature. (**b**): Reprinted with permission from Ref. [39]. Copyright 2001, Elsevier.

The kinetics of self-assembly of larger 5.6 nm PbS QDs with oleic acid (OA) ligands were studied using in-situ GISAXS by Weidman et al. [29]. GISAXS measurements of PbS_5.6_ QDs self-assembly were performed by drop casting process, although spin coated samples gave similar lattice assembly. The results extracted from GISAXS patterns showed that the PbS_5.6_ QDs self-assembled into a *bcc* superlattice starting from *fcc* orientation in the colloidal suspension and going through a body centered tetragonal (*bct*) orientation, which was likened to a Bain-like distortion. However, the superlattice evolution is not a complete Bain distortion which was also earlier reported by Bian et al., [38,39] and is depicted in Figure 3.

**Figure 4:**
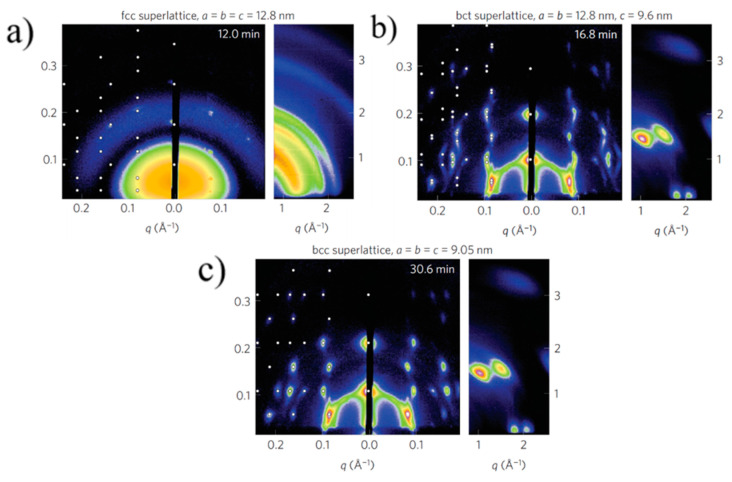
GISAXS patterns showing *fcc* (**a**) to *bcc* (**c**) transformation via an intermediate *bct* (**b**) superlattice formation. Adapted with permission from Ref. [29]. Copyright 2016, Springer Nature.

Bain distortion, commonly observed in martensite steel, involves a change in all the three lattice parameters *a*, *b* and *c* (contraction along *c* and expansion along *a* and *b*) when going from a *fcc* to *bcc* via a *bct* transformation [39]. Weidman and coworkers only observed a contraction in the length of the c axis along the [001] direction_,_ while the *a* and *b* axes remained constant. A decrease in the *c* axis implies a decreased distance between the nanocrystals, possibly due to ligand interdigitation. A contrasting result in the *c* axis contraction observed for PbS QDs with respect to other soft material systems is that the contraction reported here is at a 45° inclination with respect to the surface normal while it is rather observed normal to the surface in other soft material systems due to the vertical direction of solvent evaporation. The GISAXS patterns for the three unit cells observed during PbS_5.6_ superlattice formation are given in Figure 4.

**Figure 5:**
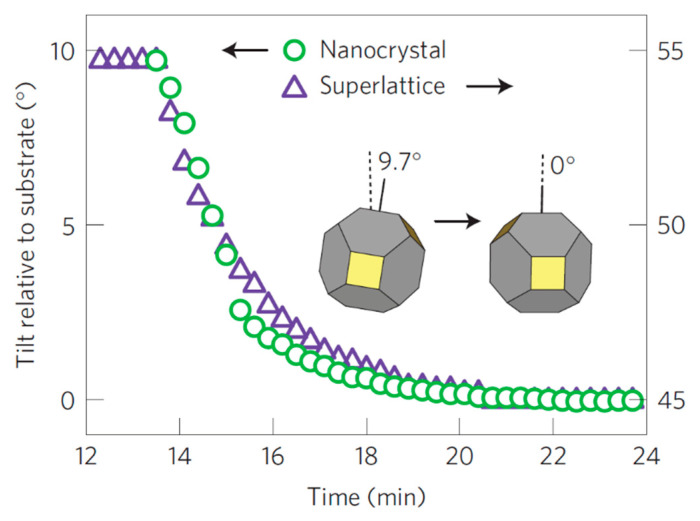
Temporal evolution of the superlattice (SL) and nanocrystal (NCs) tilt angle with respect to the substrate occurring during the fcc-to-bcc transformation in 5.6 nm PbS QDs. Adapted with permission from Ref. [29]. Copyright 2016, Springer Nature.

Another useful information extracted from the GIWAXS/GISAXS measurement of the PbS_5.6_ QDs is the change over time of the tilt angle of the nanocrystals (NC) and the superlattice (SL), relative to the substrate (Figure 5). Both SL and NCs have an exponential change of the tilt angle with time which is reported to be in accordance with the superlattice densification which is also exponential. The superlattice contracts due to the *c* axis shortening and also rotates followed by the rotation of the NCs in order to obtain the directional interaction with the neighboring NCs. This suggests that the ligands attached to the faces of the NCs drive the transformation from *fcc* to *bcc*, packing more efficiently around the NC cores, in agreement to what reported by Goodfellow et al. [40].

**Figure 6:**
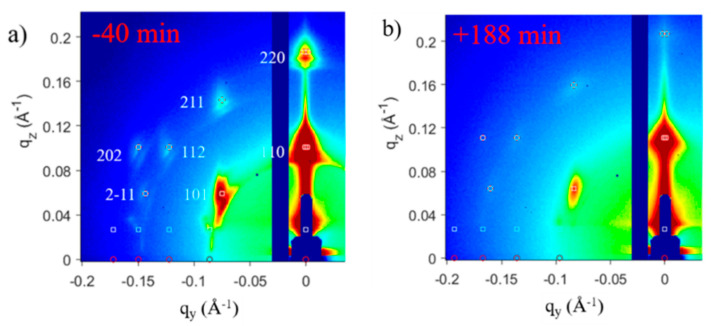
GISAXS patterns for 6.8 nm PbS QD superlattices (**a**) before and (**b**) after ligand exchange from oleic acid to tetrabutylammonium tetrathiafulvalene dicarboxylate. Time zero is defined here as the time when ligand exchange is started. Adapted with permission from Ref. [32]. Copyright 2018, American Chemical Society.

With respect to the ligands, S. Maiti et al. replaced the oleic acid (OA) surface ligands by conductive tetrabutylammonium tetrathiafulvalene dicarboxylate (TTFDA) ligands and have reported in-situ GISAXS measurements during the ligand exchange at the air–liquid interface (Figure 6) [32]. The effect of ligand exchange was measured as an additional 6% observed superlattice contraction to the initial 5% contraction occurring during self-assembly due to inter-digitization of the ligands, owing to their smaller size. The bi-dentate cross-linker (tetrathiafulvalene dicarboxylate) added, locks the nanocrystal because of its structure and size. Hence, the superlattice does not undergo further lattice contraction after ligand exchange. In this way, superlattices with the desired interparticle spacing can be achieved by freezing contraction by addition of such geometrical ligands to the nanocrystals.

**Figure 7:**
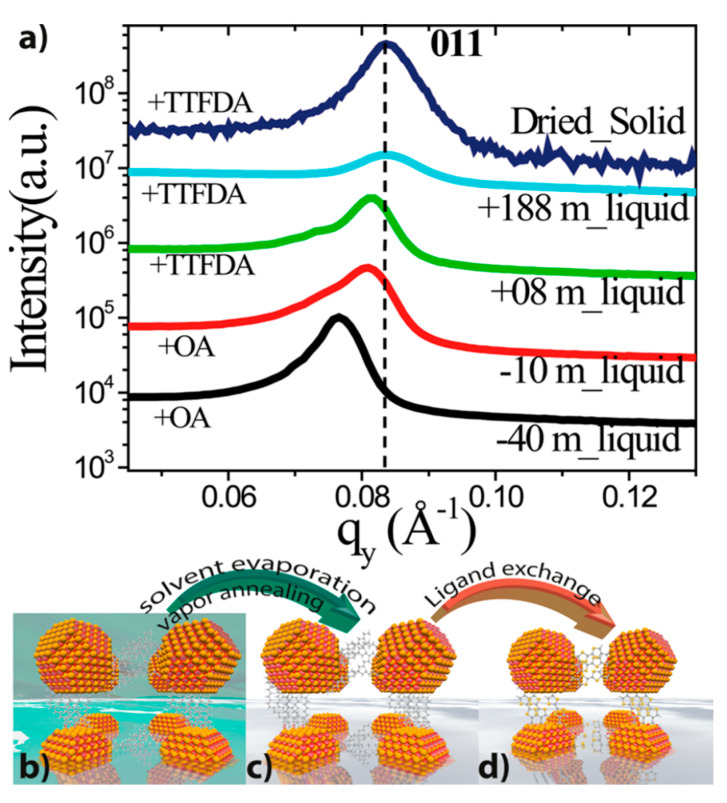
(**a**) In-plane GISAXS line profiles before and after ligand exchange, showing temporal superlattice contraction. Time zero is defined here as the time when ligand exchange is started. (**b**) Onset of superlattice while some solvent is still present (in green color). (**c**) Complete drying of NCs leads to a lateral contraction with the OA ligands as spacers. (**d**) Ligand induced contraction of the superlattice. Adapted with permission from Ref. [32]. Copyright 2018, American Chemical Society.

The change in the (011) diffraction peak observed in the in-plane GISAXS profiles (Figure 7) of the superlattice during drying suggests a clear contraction of the system while the *bcc* arrangement was retained throughout the self-assembly process.

**Figure 8:**
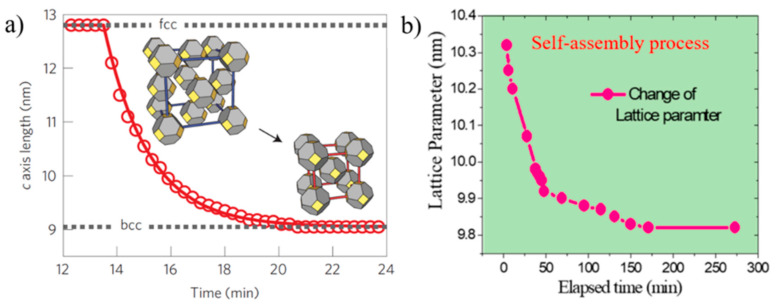
Comparison of the contraction in oleic acid (OA)-capped PbS QD assemblies during drying reported by (**a**) Weidman et al. (30%) and (**b**) S Maiti et al., (5%). (**a**): Adapted with permission from Ref. [29]. Copyright 2016, Springer Nature. (**b**): Adapted with permission. From Ref. [32]. Copyright 2018, American Chemical Society.

It is interesting to highlight some differences between the works of Weidman [29] and Maiti [32]. Figure 8 shows the comparison between the lattice contractions reported in the two works. The systems under study in these two works are PbS QDs of comparable size (5.6 nm vs 6.8 nm) and with oleic acid as surface ligand so the two works can be well compared. While Maiti et al. report a modest 5% contraction, the contraction percentage reported by Weidman et al. is about 30%. Moreover, Maiti et al. did not observe neither *fcc* nor *bct* intermediate structures, but only a *bcc* lattice along the whole self-assembly process. The main difference between this two works is that while Weidman et al. used a large angle of incidence $\alpha_{i}=0.25{}^{\circ}$ and their GISAXS measurements are more sensitive to what happens in the bulk of the drying droplet and at the liquid–substrate interface, Maiti et al. [32] performed the GISAXS measurement mostly at the liquid–air interface using $\alpha_{i}=0.15{}^{\circ}$, practically in coincidence of the system critical angle $\alpha_{i}=0.14{}^{\circ}$. It can thus be concluded that the self-assembly process at the liquid–air interface, in the bulk of the suspension and in contact with the substrate, may differ significantly. This could be the result of the different evaporation rates, capillary forces and interactions occurring in the different regions of the evolving system. This aspect should stimulate future research in this direction.

Epitaxial growth for superlattice formation of PbS QDs has been also investigated by GISAXS using a monolayer of PbS QDs serving as the substrate for the PbS QD superlattice formation [41]. Such layers allow for efficient electronic coupling, important for their application in devices [42,43]. Hence a very fundamental structural understanding is a key to fabricate heterostructures giving rise to desired properties.

**Figure 9:**
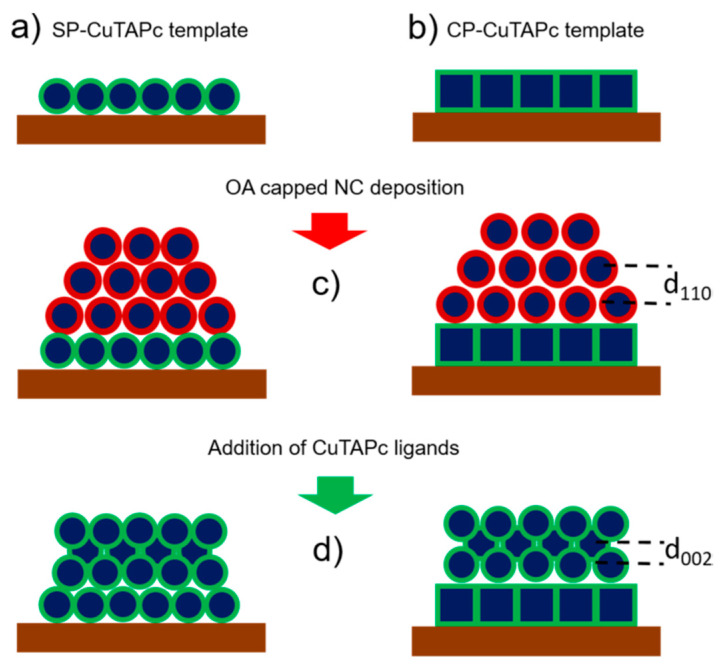
(**a**) A template of SP-CuTAPc ordered NCs and (**b**) A template of CP-CuTAPc ordered NCs. (**c**) sDeposition of OA capped NCs on the templates. (**d**) Ligand induced assembly. Reprinted with permission from Ref. [41]. Copyright 2019, American Chemical Society.

An adlayer of PbS spherical nanocrystals was deposited onto a “template” made of either hexagonal or square periodically assembled PbS NCs and they were functionalized with copper β-tetraaminophtalocyanine (CuTAPc) ligands at room temperature. Figure 9 shows a schematic of the process of ligand induced assembly, where abbreviations SP and CP stand for spherical particle and cubic particle.

**Figure 10:**
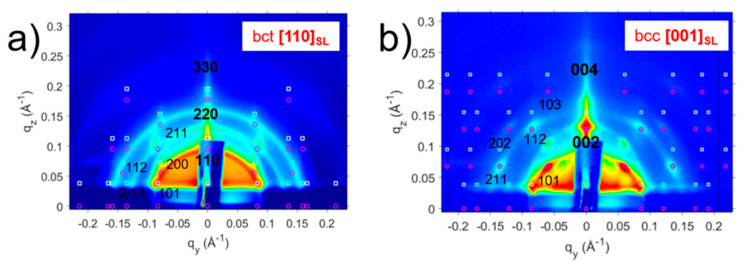
GISAXS patterns of (**a**) OA-capped PbS SP NCs deposited on an SP template showing the bct ordering with [110]_SL_ normal to the template and (**b**) ligand induced assembly for the SP-copper β-tetraaminophtalocyanine (CuTAPc) template showing the bcc ordering with [001]_SL_ normal to the template. Reprinted with permission from Ref. [41]. Copyright 2019, American Chemical Society.

First, we will examine the GISAXS and GIXD profiles of PbS NC deposition on the SP-CuTAPc template. The GISAXS patterns (Figure 10) with the oleic acid capped ligands show a *bct* ordering of the superlattice with the [110]_SL_ being perpendicular to the template. The GISAXS patterns for the ligand induced assembly has a *bcc* ordering of the superlattice with [002]_SL_ normal to the template. The GISAXS patterns have distinct and pronounced spots which prove superlattice formation of the quantum dots.

**Figure 11:**
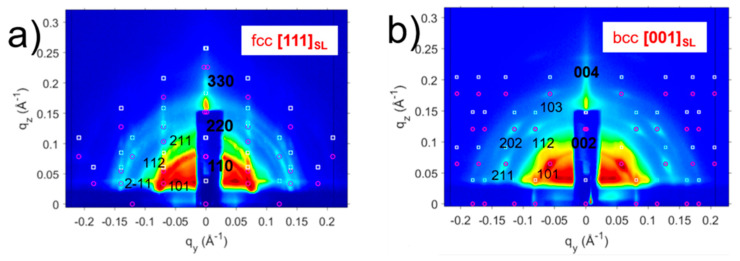
GISAXS patterns of (**a**) OA-capped PbS SP NCs deposited on a CP template showing the fcc ordering with [111]_SL_ normal to the template and (**b**) ligand induced assembly for the CP-CuTAPc template showing the bcc ordering with [001]_SL_ normal to the template. Reprinted with permission from Ref. [41]. Copyright 2019, American Chemical Society.

The authors show that the type of superlattice formed in the oleic acid capped PbS NC overlayer depends on the degree of interfacial mismatch between the template and the adlayer. A small positive mismatch value induced the *bct* superlattice formation (Figure 10a), while large negative mismatch induced *fcc* superlattice formation (Figure 11a). The two lattices transform in *bcc* structure upon the ligand exchange process, where oleic acid is exchanged for a short bifunctional (cross-linking) ligand (Figure 10b and Figure 11b). This structural change occurs via Bain-like distortion, where superlattice parameters changes and NCs rotation are triggered by the ligand exchange. All these changes can be greatly detected by the inspection of the GISAXS patterns.

**Figure 12:**
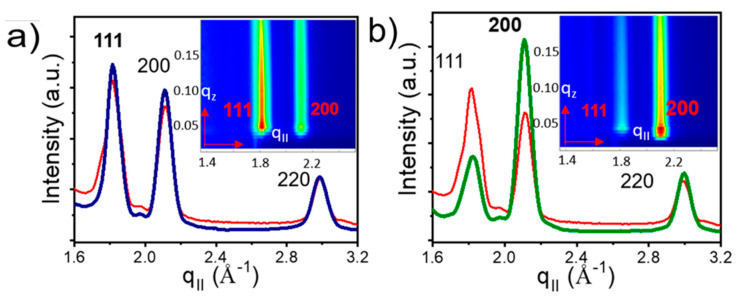
GIXD profiles of (**a**) deposited OA-capped PbS QDs with diffraction peaks observed for {111} and {200}, (**b**) ligand induced assembly for the SP-CuTAPc template where the dominant peak being {200}. Reprinted with permission from Ref. [41]. Copyright 2019, American Chemical Society.

GIXD patterns were used to investigate NC orientation inside the superlattice. The GIXD profiles for the OA capped NC superlattices grown on top of the hexagonal template show two diffraction peaks for the {111}_NC_ planes and for the {200}_NC_ planes of similar intensity (Figure 12a), suggesting no preferential atomic arrangement of the NCs. Upon ligand exchange, the 200 peak becomes dominant, indicating preferential atomic orientation induced by a strong in-plane interaction between {200}_NC_ facets induced by ligand cross-linking.

**Figure 13:**
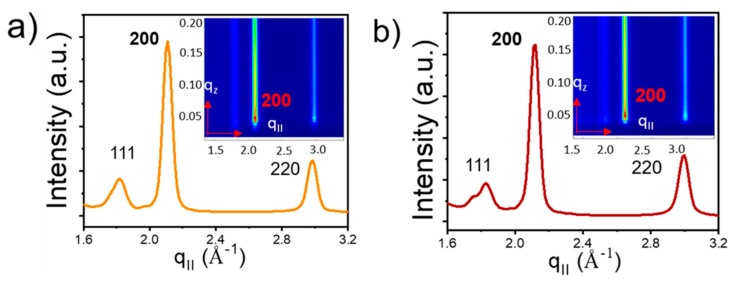
GIXD profiles of (**a**) deposited CP with oleic acid capped ligands and (**b**) ligand induced assembly for the CP-CuTAPc template. They both show the same diffraction peak of {200}. Reprinted with permission from Ref. [41]. Copyright 2019, American Chemical Society.

In contrast to the SP-CuTAPc template, the CP-CuTAPc template shows the same scattering intensity before and after the ligand exchange, with a strong signal for the {200}**_NC_** planes (Figure 13), suggesting preferential atomic orientation already before ligand exchange is performed. This orientation is retained upon ligand exchange on the square template, as the GIXD signal does not change during the exchange process. These results provide clear proof that both the nature of the template and the ligand used strongly influence the self-assembly of the nanocrystals, allowing for tune packing, orientation and inter-planar distances of the superlattices.

## 4. PbSe Quantum Dots

PbSe quantum dots are most widely used in the area of solar cells where researchers have been able to achieve power conversion efficiencies greater than 10 percent and also achieved electron mobilities of 24 cm^2^ V^−1^ s^−1^ in superlattices [44,45]. They have also been used to fabricate up conversion devices converting infra-red light to visible radiation, where these QDs were used as a sensitizing layer giving efficiencies up to 1.5 µm [46]. PbSe QDs have been shown to have a high photocurrent gain due to generation of multiple exciton generation as a filler material in a polymer based composite [47].

Moreover, PbSe QD superlattices have been shown to have high thermopower and electrical conductivity values [48]. Hence, achieving a more deeper and fundamental understanding of the self-assembly process of PbSe QDs into superlattices can indeed help engineer strategic devices with improved electrical properties [26].

Recenlty, the Vanmaekelbergh group [49] used in-situ GISAXS, GIWAXS and X-ray reflectivity (XRR) to obtain three dimensional adsorption geometries of PbSe NC monolayers at a liquid–air interface. This section will not focus on XRR, but only on GISAXS and GIWAXS. Adsorption geometries of three different sizes of PbSe NCs were studied during the last stage of the superlattice formation at the ethylene glycol-air interface.

**Figure 14:**
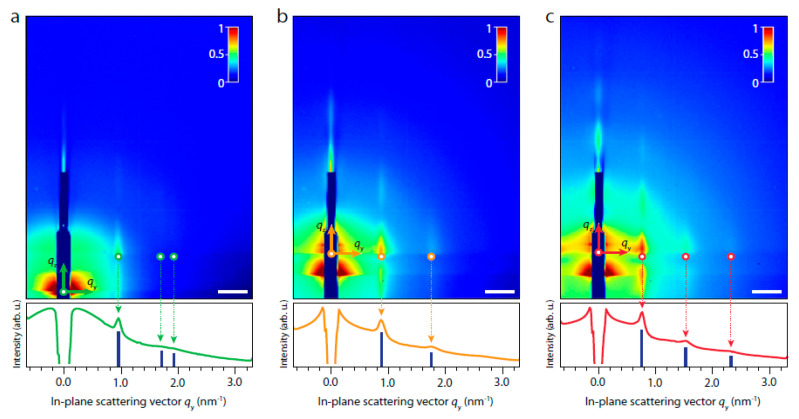
GISAXS signals for (**a**) small NCs (diameter < 5.5 nm), (**b**) medium sized NCs (diameter in the 5.5–7.6 nm range) and (**c**) large NCs (8.2–9.1 nm). Reprinted with permission from Ref. [49]. Copyright 2020, Springer Nature.

Figure 14a shows one distinct peak at $q_{y}$ = 0.94 nm^−1^ and two weak signals located at $q_{y}$ = 1.62 and 1.88 nm^−1^ for the small sized NCs (<5.5 nm). The ratio of these peaks is 1:√3:2 which suggests a 2D hexagonal lattice. On the contrary, the GISAXS patterns for the medium sized NCs and large sized NCs (>5.5 nm) show two and three Bragg rods, with a ratio of 1:2 and 1:2:3, respectively, suggesting a one dimensional ordering of the NCs.

**Figure 15:**
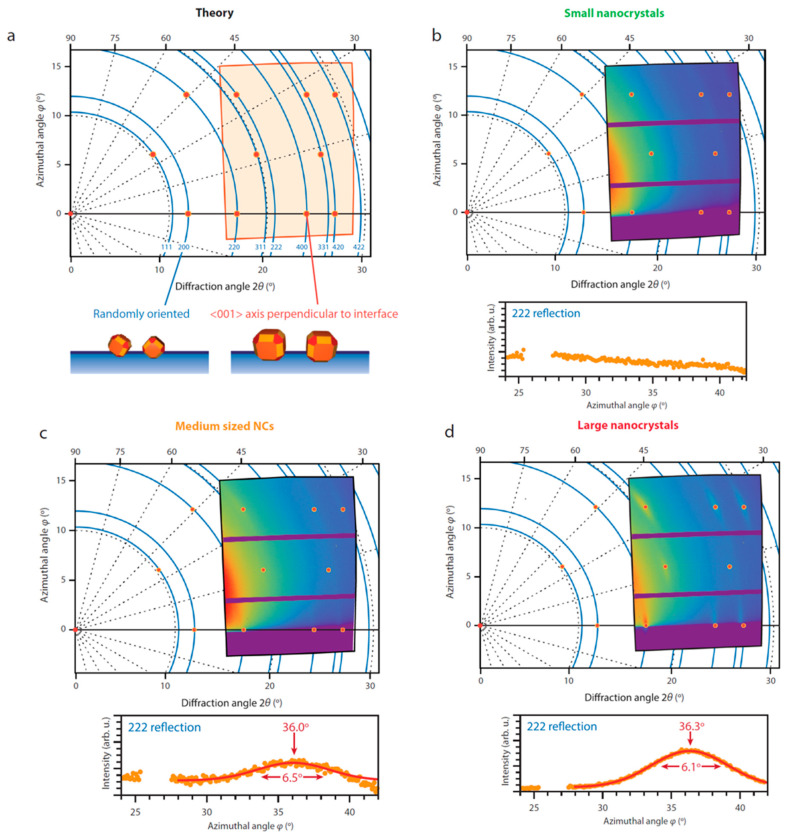
GIWAXS patterns for (**a**) no NCs, (**b**) small NCs, (**c**) medium sized NCs and (**d**) large NCs indicating the rotational degree of freedom of the NCs and the orientation of the NCs w.r.t. the liquid–air interface Reprinted with permission from Ref. [49]. Copyright 2020, Springer Nature.

Figure 15 shows the simulated and experimental GIWAXS patterns for the differently sized PbSe NCs. This is an interesting and easier way of understanding the crystallographic orientation of the PbSe NCs. As can be seen from Figure 15a, the GIWAXS pattern for a PbSe NCs lattice with their [001] axis direction oriented perpendicularly to the liquid–air interface (red dots) shows well-defined diffraction spots. If full rotational movement is allowed and the nanocrystals are isotropically oriented, the spots should become rings (blue rings). Figure 15c,d shows that the medium and large sized PbSe NCs can be well modelled with a [001] axis direction orthogonal to the liquid–air interface. The freedom of rotational motion appears to be size dependent. While large NCs have very distinct spots, the small NCs do not have such distinct spots and show powder-like diffraction. Thus, the large sized NCs have less rotational freedom compared to the medium and small sized ones. This behavior was also confirmed by analytical calculations on the adsorption geometries. The small sized NCs have small adsorption energy and small facet size and can thus easily rotate at the liquid–air interface. On the contrary, the larger the crystal size the larger the adsorption energy and the facet size, hindering the NC rotation during assembly.

**Figure 16:**
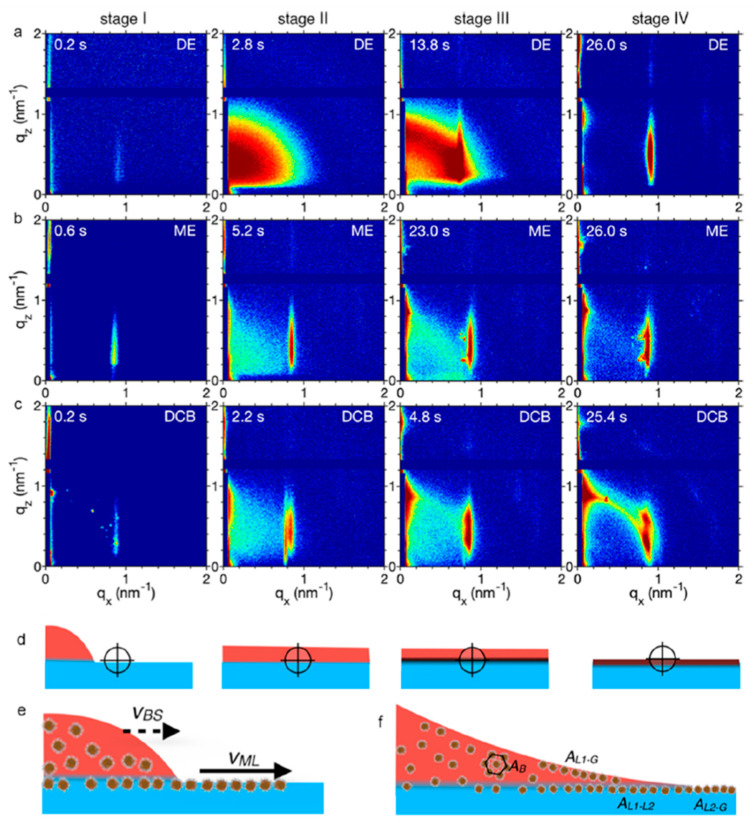
Time resolved in-situ GISAXS patterns for solvents corresponding to (**a**) decane (DE) (**b**) mesitylene (ME) and (**c**) dichlorobenzene (DCB). (**d**) Schematic of the droplet spreading, with the X-ray beam position shown by a crosshead. (**e**) Competition between the precursor film formation and droplet spreading. V_ML_ and V_BS_ represent the velocities of the bulk solution and the monolayer. (**f**) Illustration of superlattices formed at different interfaces, where A_B_ is the superlattice formed in the bulk, A_L1-G_ is the superlattice formed at the liquid–gas interface, A_L1–L2_ is the superlattice formed at the liquid–liquid interface and A_L2-G_ is the superlattice formed at the liquid–gas interface. Reprinted with permission from Ref. [50]. Copyright 2020, American Chemical Society.

Understanding droplet dynamics on the substrate is key in helping to achieve better self-assembled 2D superlattices. Balazs et al. [50] have recently explored this aspect by using the GISAXS/GIWAXS techniques and have contributed to the understanding of the role of droplet spreading during self-assembly. In this work, synchrotron based in-situ GISAXS with a temporal resolution of 200 ms has been used to study the processes happening during self-assembly of oleate capped 6 nm PbSe NCs at a liquid–liquid interface between ethylene glycol (EG) and three different solvents with decreasing NC solubility, namely decane (DE), mesitylene (ME) and dichlorobenzene (DCB). Different stages of the process could be identified (Figure 16). Stage I depicts droplets of the NC solution in contact with the EG sub-phase; stage II depicts the crossing of the droplet across the X-ray beam path which was around 1.5 cm away from the beam path during stage I; stage III shows the onset of the NC superlattice crystallization and stage IV shows the final superlattice formation. Independently of the solvent used, it can be seen from Figure 16a–c, that during stage I there is a weak signal in the GISAXS pattern which would be unexpected because the droplet is still 1.5 cm away. The resulting Bragg peak at around 0.9 nm^−1^ is due to an ordered self-assembly of PbSe NCs that has formed on the EG–air interface due to the precursor film [51]. A precursor film is formed initially because of the differences in interfacial energy at the three phase contact line. As a result, the more energetic fluid runs ahead to form a precursor film and the NPs as a result become ordered at the interface. This effect is more pronounced in ME and DCB because of the less solubility of the PbSe NCs compared to that in DE. When spreading across the X-ray beam path in stage II, DE shows a blob-like scattering pattern which suggests a dense NC solution. The system obtained from DCB shows two peaks (first and second intermittent peak) in the GISAXS pattern which have been attributed to the presence of two superlattices crystallizing one at the liquid–liquid and the other at the liquid–air interface. Another hypothesis suggested by the authors is that due to the dry and wet areas on the liquid substrate, there can be different amounts of solvation by the solvent, giving rise to two different sets of superlattices. These possibilities have been also clarified by using TEM. In stage III, the effect of the solvent volatility is observed. As the solvent evaporates, the density of the NPs increases. This increased density shows the onset of a well-ordered NC arrangement in DE, but even more pronounced in ME and DCB. Finally, stage IV shows the final superlattice formation with well-ordered arrangement for DE and ME. On the contrary, a powder ring like pattern which suggests some final degree of random orientation was observed in DCB.

## 5. Ge Quantum Dots

Germanium quantum dots are also a matter of intensive research for their use in optoelectronic devices [52]. Zhou et al. [53] have shown strong electronic coupling between adjacently placed Ge QDs and mentioned the importance of having control over the positioning of the QDs to achieve strong coupling for applications such as LEDs [52] and photodetectors [54]. Hence, it becomes important to know the self-assembly process of Ge QDs.

**Figure 17:**
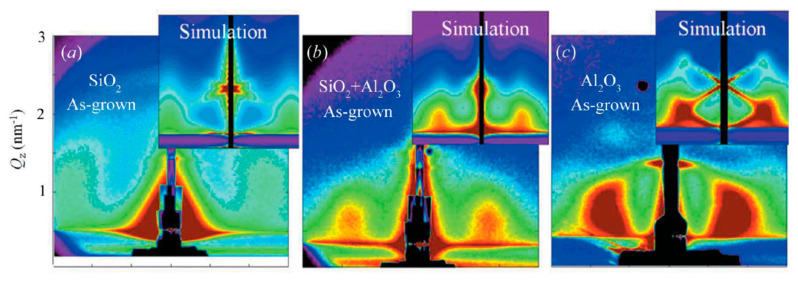
GISAXS experimental patterns together with simulations for Ge QDs deposited as multilayered films in (**a**) silica, (**b**) mullite and (**c**) alumina. Reproduced with permission from Ref. [55]. Copyright 2013, International Union of Crystallography.

M. Buljan et al. [55] used GISAXS to obtain a fundamental understanding of the ordering type, the degree of regularity and the effect of the matrices into which Ge QDs were self-assembled by magnetron sputtering deposition technique. As can be seen from Figure 17, there are differences in the number and the width of the GISAXS peaks which naturally suggest that the degree of ordering of Ge QDs is different in different matrices. Ge QDs deposited on SiO_2_ and mullite matrixes show two symmetric GISAXS peaks around the *y*-axis (Figure 17a,b), whereas there are four peaks visible for the Ge QDs embedded in the alumina matrix (Figure 17c). GISAXS simulations show that the [111] axis set is perpendicular to the substrate, suggesting that the multilayer growth happens perpendicularly to the substrate.

**Figure 18:**
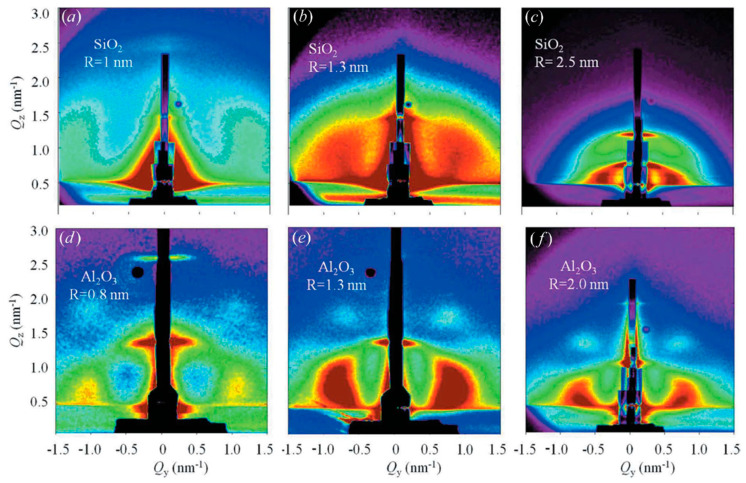
GISAXS patterns for multilayer films with different size of Ge QDs deposited in silica (**a**–**c**) and alumina (**d**–**f**) matrices. Reproduced with permission from Ref. [56]. Copyright 2019, International Union of Crystallography.

For the silica matrix, Figure 18 shows that the largest quantum dots exhibit the best ordering quality as evident by the distinct peaks. This is a helpful result for Ge to be used in device applications and in nanotechnology because larger quantum confinement is observed with larger sizes of Ge QDs [16]. As a consequence, superlattices of larger Ge QDs have less disorder and better quantum confinement.

However, in order to draw clearer conclusions of the effect of the QD size on the ordering quality, more experiments need to be performed. There may be some size dependent electronic properties which may create a variation in the ordering type in the particular matrix. These experiments should be carried out in the future to highlight a clear trend with size which can be generalized upon.

**Figure 19:**
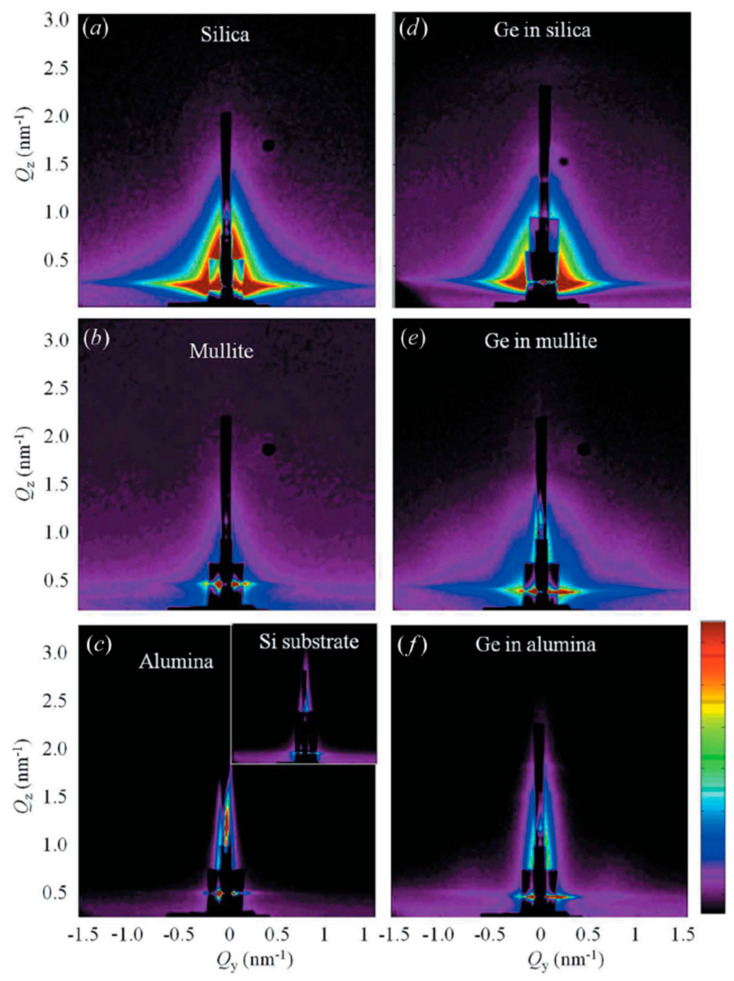
GISAXS patterns at the surface of the different matrices (**a**–**c**) before and (**d**–**f**) after Ge QD deposition. The lower diffused scattering intensity for Ge/alumina suggests higher degree of ordering and low surface roughness. Reproduced with permission from Ref. [55]. Copyright 2013, International Union of Crystallography.

GISAXS results show also that amorphous alumina is the best matrix for efficient Ge QD self-assembly, resulting in minimal disordering observed in the GISAXS patterns for alumina (Figure 19**)**. Hence, it is clearly shown that multilayered thin films with different matrices with self-assembling quantum dots have different structural ordering.

**Figure 20:**
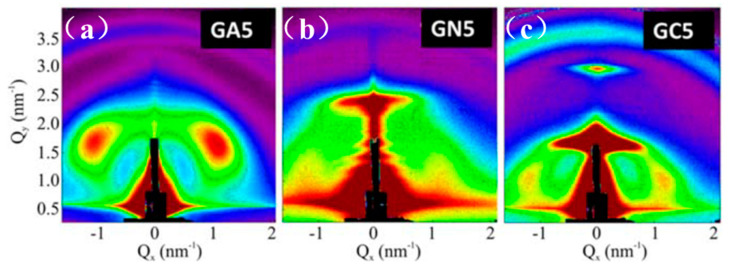
GISAXS patterns showing a 3D ordering of Ge QD superlattices in the different matrices of (**a**) A (Al2O3), (**b**) N (Si3N4) and (**c**) C (SiC) at a temperature of 500 °C. Reproduced with permission from Ref. [56]. Copyright 2019, International Union of Crystallography.

Matrix dependency on the structural ordering of Ge QDs has also been studied by Nekic et al., [56] showing the formation of three dimensional ordered Ge QD lattices in alumina, silicon nitride and silicon carbide based matrices. Ge QDs were deposited in different matrices by magnetron sputtering at temperatures ranging from 300 to 800 °C with an increment of 100 °C. GISAXS patterns of Ge QDs deposited at the 500 °C are shown in Figure 20 and exhibit the most pronounced Bragg spots amongst the tested lower temperatures.

**Figure 21:**
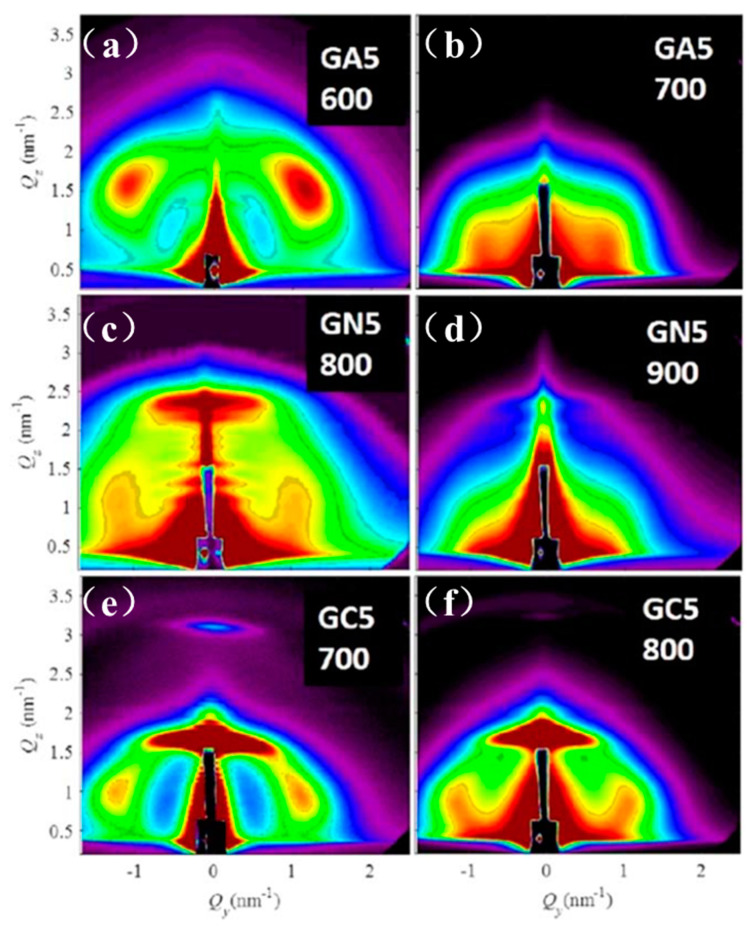
GISAXS patterns of annealed films showing the collapse of the ordering of QDs at high temperature (**a**–**f**). Reproduced with permission from Ref. [56]. Copyright 2019, International Union of Crystallography.

Collapse of the ordering of the respective Ge QD lattices in different matrices was inferred from strong alteration of the GISAXS patterns and subsequent loss of the Bragg signals at high temperature (Figure 21). Thus, temperature resolved GISAXS helps to know the optimum range of annealing temperatures when fabricating such systems.

**Figure 22:**
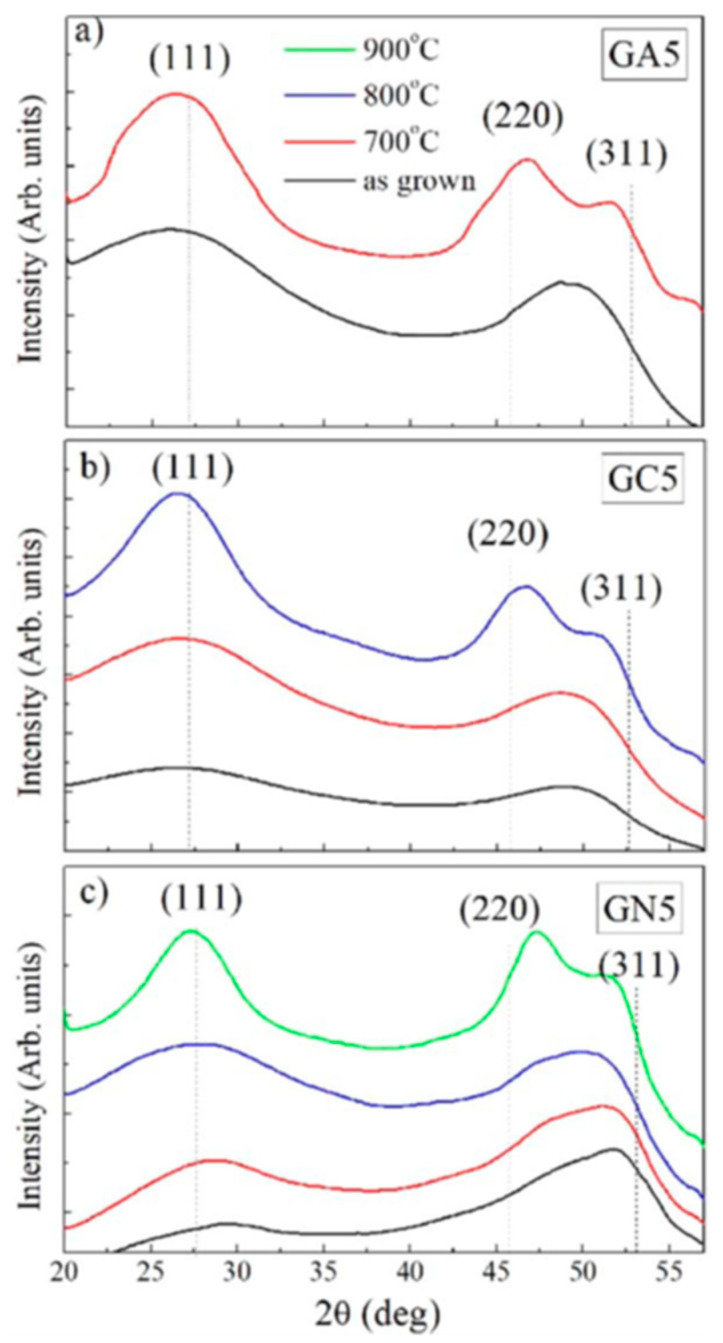
GIWAXS line profiles of Ge QD films deposited in (**a**) Al_2_O_3_, (**b**) SiC and (**c**) Si_3_N_4_ matrices and annealed at different temperatures (700, 800 and 900 °C). Reproduced with permission from Ref. [56]. Copyright 2019, International Union of Crystallography.

Moreover, the authors performed a comparative study of the crystalline properties determined by the GIWAXS technique (Figure 22). GIWAXS profiles of the as-deposited multilayers show only two broad peaks which correspond to amorphous Ge QDs. [57] Sharpening of the 111 peak and appearance of well separated 220 and 311 peaks notify crystallization of the QDs. Interestingly, the observed crystallization temperatures correspond to the temperature for the collapse of the QD superlattices.

From the examples reported above it is evident that a clear influence of the substrate temperature on self-assembly and crystallinity of magnetron sputtered Ge QDs can be well assessed by GISAXS and GIWAXS. We believe that in-situ GISAXS and GIWAXS during the magnetron sputtering of these systems can bring further useful information for technological applications of the Ge QD lattices.

## 6. Fe_2_O_3_ Quantum Dots

Iron oxide nanocrystals are well studied materials. They have been shown to be useful for photo–electro–chemical (PEC) water splitting systems, [58] high performance Li-ion batteries when embedded in nitrogen-doped carbon networks, [59] neuronal manipulations by using a nanocomposite of gamma Fe_2_O_3_/Nitrogen doped carbon dots, [60] and also for selective fluorescence sensing of Hg(II) by using gamma Fe_2_O_3_ colloidal quantum dots as nanoprobes [61]. Most importantly, ordered assemblies of these particles are required for optoelectronic device applications such as in spin valves [62] and for photonic devices [63], which both include self-assembled 1D arrays or 2D and 3D structures. Hence, an understanding into the structural ordering process would open doors to improve the assembled structure of these quantum dot systems.

**Figure 23:**
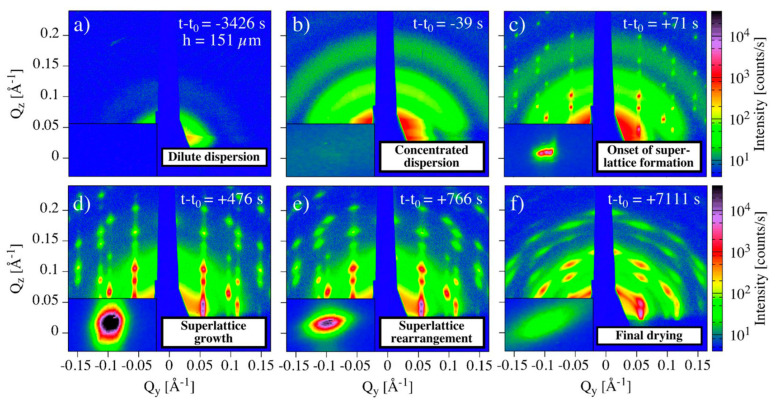
(**a**–**f**) Time resolved in-situ GISAXS of self-assembled Fe_2_O_3_ QDs during slow evaporation from toluene, showing the evolution of a 3D *fcc* superlattice from a disordered to an ordered superlattice. Reprinted with permission from Ref. [27]. Copyright 2017, Springer Nature.

The mechanism of formation of oleic acid capped Fe_2_O_3_ nanocrystals of 9.9 nm diameter drop casted from toluene was studied by in-situ GISAXS. [27] A transition from a disordered system made of dispersed colloids in solution into a highly ordered superlattice arrangement with a rhombohedral structure was observed (Figure 23). It was reported that the build-up of capillary pressure at the interface between the saturated and partially saturated regions of the receding droplet promotes rapid formation of highly ordered superlattices. During controlled slow evaporation, the structure contracts, converging to a nearly perfect *fcc* lattice. The images are labelled for the onset, growth and rearrangement of superlattices. Onset of superlattice formation was set as the first moment of appearance of sharp Bragg peaks on the GISAXS pattern (Figure 23c). Interestingly, these Bragg peaks are immediately sharp, indicating that the domain size of the superlattice is large from their first time of appearance. However, when has the nucleation of the superlattice begun? Unfortunately, the time resolution used in this work was not enough to investigate this aspect. For a longer drying time, the Bragg peaks broadened and diffused out along arcs. This can be attributed to the fact that there is a tilt of the superlattice relative to the substrate.

**Figure 24:**
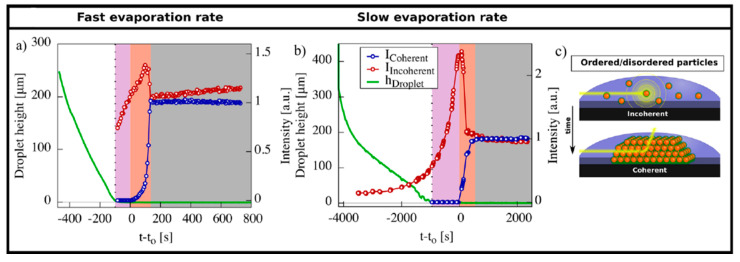
Time dependent GISAXS scattering intensity recorded during (**a**) fast and (**b**) slow drying of Fe_2_O_3_ quantum dots from toluene. The graphs show the coherent (from ordered structures) and incoherent (from disordered assembly) scattering intensities together with the droplet height evolution during fast and slow drop casting. (**c**) Transition from an incoherent to coherent scattering with time due to formation of an ordered superlattice. Reprinted with permission from Ref. [27]. Copyright 2017, Springer Nature.

Figure 24 shows the existence of different stages occurring during drying both for fast and slow evaporation. In the first stage (white zone in Figure 24), a dilute dispersion of nanoparticles can be observed. Authors report that the droplet height decreases linearly for both the fast and slow evaporation systems, whereas it is clearly visible that there is some non-linearity in the curve for the slow evaporation rate. However, as the dilute dispersion phase shows no considerable scattering, this effect can be neglected. Next, in the concentration dispersion stage highlighted by the light purple region, an increase in the incoherent diffuse scattering occurs (disordered particles), but no coherent scattering from ordered particles is recorded. A steep rise of the coherent and incoherent scattering intensities is instead recorded in the region of the onset of superlattice formation (pink color) which is obvious from the fact that the concentration of the nanospheres is increasing. Generally, it was found that the quality of the superlattices was higher when slow, controlled evaporation of the solvent was used.

**Figure 25:**
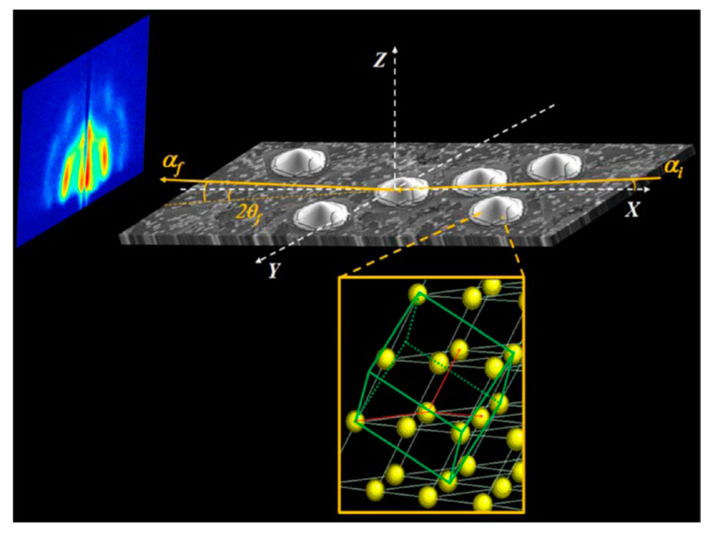
Schematic of the GISAXS scattering geometry and sample morphology for ordered 3D Fe_2_O_3_ superlattice islands. The inset shows the Fe_2_O_3_ NC (yellow spheres) arrangement within a surface protruding island. Reprinted with permission from Ref. [64]. Copyright 2012, American Chemical Society.

**Figure 26:**
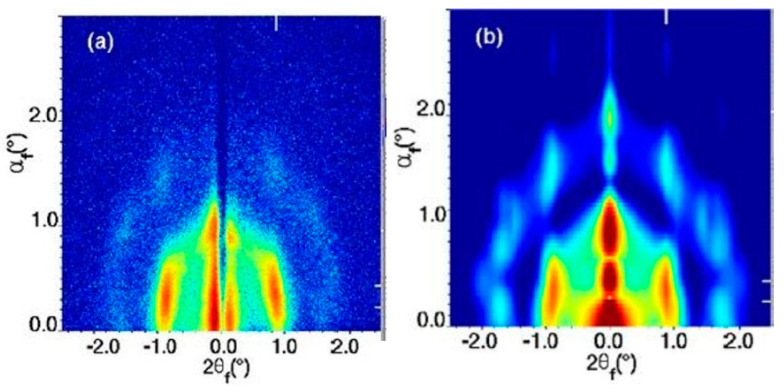
GISAXS patterns for (**a**) experimental and (**b**) simulated in the first order Born Approximation. Reprinted with permission from Ref. [64]. Copyright 2012, American Chemical Society

**Figure 27:**
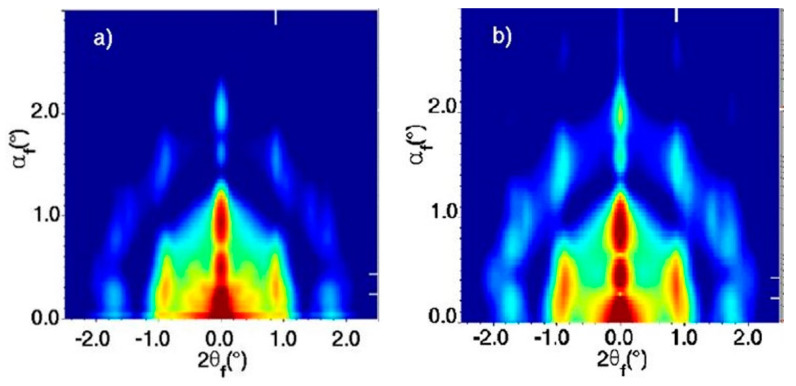
Comparing the (**a**) Distorted Wave Born Approximation (DWBA) and (**b**) kinetic approximation (or first order Born Approximation) simulated GISAXS patterns. Reprinted with permission from Ref. [64]. Copyright 2012, American Chemical Society.

When dealing with GISAXS data, careful considerations about data simulation and interpretation also need to be made, depending on the chosen incident angle. Normally, Distorted Wave Born Approximation (DWBA) has to be taken into account to simulate correctly the experimental patterns [64]. However, this is not necessarily the case when very small incident angles are used and the QDs self-assemble in well-developed 3D structures protruding higher than a flat substrate. Islands consisting of 3D highly ordered superlattices of iron oxide nanocrystals obtained by magnetic field assisted self-assembly were investigated by GISAXS by D. Altamura et al. (Figure 25**)** [65]. In this case, the authors show that the best GISAXS patterns are obtained when a very small incident angle of 0.05° is used. In this case, the GISAXS patterns can be successfully simulated using the classical Born approximation for transmission scattering, as multiple scattering events considered in the DWBA can be neglected at such small incident angles (Figure 26). On the contrary, when DWBA was used, additional scattering intensity at low scattering angles was observed, while not present in the real data (Figure 27).

## 7. Conclusions

In this review we have presented many recent examples from various research works wherein authors have used GISAXS/GIWAXS (or GIXD) techniques to understand the formation and structure of superlattices of quantum dots. The examples discussed here clearly show the potential of GISAXS and GIWAXS to study not only the structural details of the superlattices in a static manner, but most importantly the kinetic parameters during the superlattice evolution process. Key parameters such as interparticle distances, lattice parameters, tilt of lattice planes and tilt of particles as well as degree of lattice/particle alignment can all be obtained by GISAXS/GIWAXS analysis statically or dynamically, over time, temperature, vapor exposure etc. The evolution of these parameters with time and the extent of how much they change during the superlattice formation is a function of the processing conditions (i.e., evaporation rate), of the surface energies in the game (surface tensions and adsorption energies at the interfaces) and of the quantum dot size.

Even more relevant for the field of quantum dots assembly, the examples provided here show how GISAXS/GIWAXS can be employed to follow the structural transformations associated to the ligand exchange process. These experiments can be performed both at the solid–liquid and at the liquid–air interfaces. We believe that this particular application will enable scientific breakthrough in QD science, as it can provide valuable information on ligand conformation, ligand affinity towards the nanocrystals and anisotropic distribution/interaction.

Moreover, GISAXS/GIWAXS have also been used to study the growth of binary superlattices, which are another kind of superlattice, giving rise to interesting properties. [66,67,68] It is also important to mention that combining results coming from other techniques such as XRR, TEM, SEM and AFM with observations from GISAXS/GIWAXS can provide a very in-depth information about the thermodynamic and kinetics of the QD self-assembly process. We thus foresee that the use of GISAXS/GIWAXS, the development of specific mathematical models to simulate experimental patterns and coupling with macroscopic property measurements and other complementary techniques will keep increasing in the years to come, contributing to the increase in knowledge in QD science.
